# Determination of Acetamiprid Residues in Vegetables by Indirect Competitive Chemiluminescence Enzyme Immunoassay

**DOI:** 10.3390/foods11162507

**Published:** 2022-08-19

**Authors:** Zixin Zhu, Qiuyun Shi, Jianwei Wu, Kangli He, Jianguo Feng, Sa Dong

**Affiliations:** School of Horticulture and Plant Protection, Yangzhou University, Yangzhou 225009, China

**Keywords:** acetamiprid, chemiluminescence enzyme immunoassay, rapid detection, residue, vegetable

## Abstract

Acetamiprid (ACE) is widely used in various vegetables to control pests, resulting in residues and posing a threat to human health. For the rapid detection of ACE residues in vegetables, an indirect competitive chemiluminescence enzyme immunoassay (ic-CLEIA) was established. The optimized experimental parameters were as follows: the concentrations of coating antigen (ACE-BSA) and anti-ACE monoclonal antibody were 0.4 and 0.6 µg/mL, respectively; the pre-incubation time of anti-ACE monoclonal antibody and ACE (sample) solution was 30 min; the dilution ratio of goat anti-mouse-HRP antibody was 1:2500; and the reaction time of chemiluminescence was 20 min. The half-maximum inhibition concentration (IC_50_), the detection range (IC_10_–IC_90_), and the detection limit (LOD, IC_10_) of the ic-CLEIA were 10.24, 0.70–96.31, and 0.70 ng/mL, respectively. The cross-reactivity rates of four neonicotinoid structural analogues (nitenpyram, thiacloprid, thiamethoxam, and clothianidin) were all less than 10%, showing good specificity. The average recovery rates in Chinese cabbage and cucumber were 82.7–112.2%, with the coefficient of variation (CV) lower than 9.19%, which was highly correlated with the results of high-performance liquid chromatography (HPLC). The established ic-CLEIA has the advantages of simple pretreatment and detection process, good sensitivity and accuracy, and can meet the needs of rapid screening of ACE residues in vegetables.

## 1. Introduction

Acetamiprid (ACE), a new kind of chlorinated nicotinoid insecticide, with strong contact and stomach toxicity, as well as excellent internal absorption activity, is widely used in the control of aphid, whitefly, thrips and other pests on vegetables because of its quick insecticidal effect, low dosage, high activity, wide insecticidal spectrum and long duration [1,2]. Although ACE is a low-toxicity pesticide, current studies have shown that it has certain genotoxicity and cytotoxicity, has adverse effects on the nervous system and reproductive system of animals, and poses a threat to human health [3,4,5,6,7,8,9]. To limit its use, national and international organizations established maximum residue levels (MRLs) for ACE. In accordance with Annex II of Regulation (EC) No 396/2005, the MRLs of ACE in Chinese cabbage (code number: 243010) and cucumber (code number: 232010) were 1.5 and 0.3 mg/kg, respectively [10]. According to China’s national standards (GB 2763-2021), the MRLs of ACE in different vegetables are 0.02–5 mg/kg (https://www.sdtdata.com/fx/fmoa/tsLibCard/183688.html, accessed on 18 May 2022). Therefore, it is of great significance to monitor ACE residual levels in vegetables to ensure food safety.

At present, the reported methods for detecting ACE residues are mainly focused on instrumental methods, such as high-performance liquid chromatography (HPLC), gas chromatography (GC), and chromatography–mass spectrometry (GC–MS) [11,12,13,14,15]. The instrument methods have high sensitivity, high accuracy, and good selectivity, but the equipment is expensive and requires professional laboratory personnel to operate, and the operation is complex and time-consuming, so it is not suitable for on-site rapid detection of ACE residues. The immunoassay method is simple, rapid, and can detect a large number of samples in a short time, which can be used for high-throughput detection of samples in the field [16]. At present, there are few studies on the use of chemiluminescence enzyme immunoassay for ACE detection, combining a highly specific immune response with a highly sensitive chemiluminescence reaction, which can increase the sensitivity by 2–3 fold compared with the traditional ELISA method [17].

In this study, a highly sensitive indirect competitive chemiluminescence enzyme immunoassay (ic-CLEIA) for ACE detection was established based on anti-ACE monoclonal antibody by optimizing the concentration of coating antigen and antibody, the pre-incubation time of anti-ACE monoclonal antibody and ACE (sample) solution, the dilution ratio of goat anti-mouse-HRP antibody and chemiluminescence reaction time. The ic-CLEIA was then evaluated by recovery experiment with simple sample pretreatment, which showed that the method was suitable for the detection of ACE in real samples. This study provides technical support for the rapid detection of ACE residues in vegetables, and has certain reference value for the detection of other pesticide residues.

## 2. Materials and Methods

### 2.1. Materials, Reagents and Equipment

Chinese cabbages and cucumbers (commercially available). Anti-ACE monoclonal antibody (anti-ACE mAb, 1.23 mg/mL) and coating antigen (ACE-BSA, 2.2 mg/mL) were acquired from Shandong Lvdu Bio-sciences and Technology Co., Ltd. (Binzhou, China). ACE standard (>99%) was purchased from Tiperi Instrument Equipment Co., Ltd. (Nanjing, China). Goat anti-mouse-HRP antibody was purchased from KPL Inc. (Gaithersburg, MD, USA). Skim milk were provided by Solarbio Science and Technology Co., Ltd., (Beijing, China). SuperSignal^TM^ ELISA Pico Chemiluminescent Substrate kit were purchased from Thermo Fisher Scientific (Thermo, Waltham, MA, USA). HPLC-grade acetonitrile was purchased from Tedia Company Inc. (Fairfield, OH, USA). The 96-well white plates were purchased from Corning Inc. (Corning, NY, USA). All other reagents and chemicals used were of analytical grade.

ACE standard solution: 0.05 g ACE standard was dissolved in 50 mL methanol to make a solution of 1 mg/mL. CBS solution: 2.93 g NaHCO_3_ and 1.59 g Na_2_CO_3_ were weighed and dissolved in double distilled water, constant volume to 1 L, pH 9.6. PBS: 0.2 g KCI, 8.0 g NaCl, 2.9 g Na_2_HPO_4_·12H_2_O and 0.2 g K_2_HPO_4_ were weighed and dissolved in double distilled water, constant volume to 1 L, pH 7.4. PBST: 0.5 mL Tween-20 was added to 1 L PBS. 3% MPBS: 0.3 g skim milk was weighed and dissolved in 10 mL PBS.

Electrothermal constant temperature precision incubator was purchased from Taisite Instrument Co., Ltd. (Tianjin, China). Multimode reader was purchased from Thermo Fisher Scientific (Thermo, Waltham, MA, USA). Highspeed freezing centrifuge was purchased from Eppendorf Inc. (Hamburg, Germany). LC-2000 high-performance liquid chromatograph was purchased from Hitachi Ltd. (Tokyo, Japan). Mili-Q ultrapure water machine was purchased from Millipore Ltd. (Burlington, MA, USA).

### 2.2. The Procedure of ic-CLEIA

First, a 96-well white plate was coated with the coating antigen (100 μL/well) in CBS at 37 °C for 2 h. After washing the plate with PBST (300 μL/well) three times, it was closed with 3% MPBS (200 μL/well) at 37 °C for 2 h. After washing the plate with PBST (300 μL/well) three times, the pre-incubation solution (diluted anti-ACE monoclonal antibody with PBS, mixed 50 μL antibody solution with 50 μL ACE solution, pre-incubated at 37 °C for a certain time) was added to the 96-well white plate and incubated at 37 °C for 1 h. After washing the plate with PBST (300 μL/well) three times, it was incubated with goat anti-mouse-HRP antibody (100 μL/well) in PBS at 37 °C for 1 h. Finally, equal proportions of chemiluminescence solution A and solution B in the SuperSignal^TM^ ELISA Pico Chemiluminescent Substrate kit (100 μL/well) were added to the 96-well white plate. After incubation in the dark for a certain time, the luminescence value RLU was measured.

### 2.3. Optimization of ic-CLEIA

According to the previous results of indirect competitive enzyme-linked immunosorbent assay (ELISA) and checkerboard titration, the initial concentration of coating antigen was 0.37 µg/mL. The initial concentration of anti-ACE monoclonal antibody was 0.15 μg/mL. The pre-incubation time of anti-ACE monoclonal antibody and ACE (sample) solution was 20 min. The dilution ratio of goat anti-mouse HRP antibody was 1:5000. The chemiluminescence reaction time was 10 min.

Then, to improve the detection performance of ic-CLEIA, the effects of the coating antigen concentration (0.1, 0.2, 0.4, 0.8, and 1.6 μg/mL), antibody concentration (0.0375, 0.075, 0.15, 0.3, 0.6, and 1.2 µg/mL), the pre-incubation time of anti-ACE monoclonal antibody and ACE (sample) solution (10, 20, 30, and 40 min), the dilution ratio of goat anti-mouse-HRP antibody (1: 625, 1:1250, 1:2500, and 1:5000), and chemiluminescence reaction time (5, 10, 15, 20, 25, 30, and 35 min) on the sensitivity of ic-CLEIA were investigated by single factor experiment. According to the procedure of ic-CLEIA, standard curves for ACE detection using ic-CLEIA under each condition were established, and RLUmax (luminescence value without ACE) and the IC_50_ (half maximal inhibitory concentration, ACE concentration at 50% competitive inhibition) were calculated based on the standard curves. The RLUmax/IC_50_ ratio was used to evaluate the influence of specific factors on the detection performance of ic-CLIEA, and the higher the ratio, the higher the sensitivity under this condition [18]. The optimal reaction conditions of ic-CLEIA were high RLUmax/IC_50_, a low IC_50_ and moderate RLUmax.

### 2.4. Establishment of the Standard Curve for ic-CLIEA

Under optimal conditions, the standard curve was drawn with ACE concentration (200, 100, 50, 25, 12.5, 6.25, 3.125, 1.5625, 0.8, 0.4, 0.2, and 0.1 ng/mL) as the abscissa and B/B0 (B is the RLU value with ACE and B0 is the RLU value without ACE) as the ordinate. Finally, the IC_50_, the detection range (determined according to the standard curve), and the limit of detection (LOD, IC_10_) of the ic-CLIEA method were calculated according to the standard curve.

### 2.5. Specificity of ic-CLIEA

The cross-reactivity rate was used to evaluate the specificity of ic-CLIEA method, and the higher the cross-reactivity rate, the worse the specificity. In this study, four neonicotinoid structural analogues (nitenpyram, thiacloprid, thiamethoxam, and clothianidin) were selected and determined by the established ic-CLIEA, then the IC_50_ and the cross-reactivity rates (CR%, CR% = IC_50_ of ACE/IC_50_ of analogue × 100) were calculated to evaluate the specificity of ic-CLIEA.

### 2.6. Sample Pretreatment

The Chinese cabbage and cucumber samples purchased from a supermarket were first confirmed by HPLC, and the samples without ACE residue were used for the recovery experiments.

The sample pretreatment used for ic-CLIEA detection was simplified based on previously reported methods [19]. Briefly, 100 g samples were homogenized and then the juice was squeezed out. A volume of 2 mL 99.5% acetone was added to the juice and left to stand for 5 min, then filtered with filter paper and the filtrate was collected. The filtrate was centrifuged at 6000 rpm for 5 min to obtain the supernatant, and the volume was fixed to 30 mL with sub-boiling water. The solution was passed through a 0.22 µm filter membrane and then determined by ic-CLIEA.

The sample pretreatment used for HPLC detection was performed according to the previously reported method with minor modification [20]. Briefly, 2 g samples were weighed and cut into 50 mL centrifuge tubes, then 10 mL acetonitrile was added to each centrifuge tube, and the samples were sonicated for 10 min. An appropriate amount of sodium chloride and anhydrous magnesium sulfate was added to the above mixture, and the mixture was vortexed and oscillated for 5 min, then centrifuged at 5000 rpm for 5 min. After centrifugation, 150 mg anhydrous magnesium sulfate and 50 mg primary secondary amine (PSA) were added to 1.5 mL of the above supernatant, thoroughly mixed by shaking, and centrifuged at 10,000 rpm for 5 min. The supernatant was passed through a 0.22 μm filter membrane and used for HPLC detection.

### 2.7. Elimination of Matrix Interference

A dilution method is usually used to eliminate matrix interference. A blank matrix without ACE is treated according to the sample pretreatment method during ic-CLIEA detection, and the filtrate is diluted with PBS and used to prepare ACE solution of serial concentration. Subsequently, ic-CLIEA was used for determination, and the standard curves were drawn, respectively. The influence of sample matrix on ACE detection was analyzed by comparing the above standard curve with the standard curve drawn by using PBS to prepare ACE solution without matrix.

### 2.8. Recovery Experiments

Since ACE is mostly used in Chinese cabbage, cabbage, cucumber and tomato, Chinese cabbage and cucumber were selected as actual samples in this experiment.

ACE standard solution was added to the blank samples to make the ACE content 1.5, 6, 30 μg/kg. After sample pretreatment, the established ic-CLIEA and HPLC methods were used for determination, and the recovery rate and the coefficient of variation (CV) were calculated.

HPLC conditions: Hypersil ODS (4.6 mm × 250 mm, 5 μm). The mobile phase was acetonitrile:water = 30:70 (V1:V2) and the flow rate of the mobile phase was 1.0 mL/min. The UV detection wavelength was 250 nm, the column temperature was 30 °C, and the injection volume was 5 μL.

### 2.9. Data Analysis

When the ic-CLIEA method was used for determination, the ACE concentration in the sample was calculated from the standard curve according to the RLU value, and then multiplied by the corresponding dilution ratio, which was the actual concentration of ACE in the sample. All experiments were repeated three times, and all the data in the results were the average values of the measured data.

## 3. Results

### 3.1. Optimization of Coating Antigen Concentration

**Figure 1:**
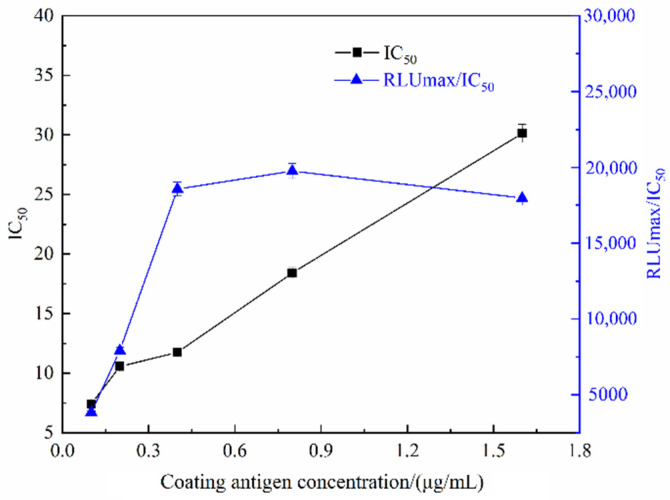
The IC_50_ and RLUmax/IC_50_ of ic-CLIEA under different coating antigen concentrations for ACE detection.

Under the fixed concentration of antibody, the luminescence intensity and sensitivity increased with the increase in the coating antigen concentration, but when the concentration was too high, the steric hindrance increased, and the luminescence intensity and sensitivity decreased [18]. Therefore, it is necessary to optimize the concentration of coating antigen. The coating antigen was diluted to 0.1, 0.2, 0.4, 0.8, and 1.6 µg/mL and then determined by ic-CLIEA, respectively. The influence of the coating antigen concentration on the detection sensitivity and RLUmax/IC_50_ was analyzed. The results showed that RLUmax/IC_50_ increased firstly and then decreased with the increase in the coating antigen concentration. When the coating antigen concentration was 0.4 and 0.8 μg/mL, the values of RLUmax/IC_50_ were larger, and the IC_50_ gradually increased with the increase in coating antigen concentration. According to the principle that RLUmax/IC_50_ should be as large as possible and the IC_50_ should be as small as possible, the optimal concentration of coating antigen was selected as 0.4 μg/mL (Figure 1).

### 3.2. Optimization of Antibody Concentration

**Figure 2:**
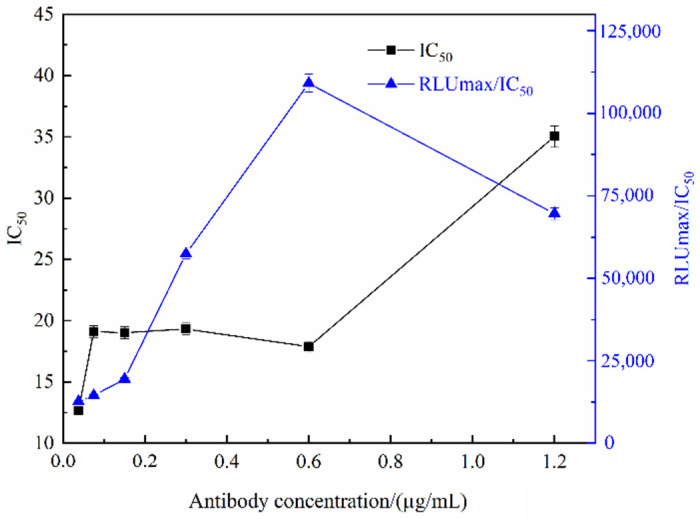
The IC_50_ and RLUmax/IC_50_ of ic-CLIEA with different antibody concentrations for ACE detection.

The 96-well white plate was coated with the optimal concentration of coating antigen, and anti-ACE monoclonal antibody was diluted to 0.0375, 0.075, 0.15, 0.3, 0.6, and 1.2 µg/mL, followed by ic-CLIEA assay. The influence of antibody concentration on the detection sensitivity and RLUmax/IC_50_ was analyzed. As shown in Figure 2, with the increase in antibody concentration, the RLUmax/IC_50_ increased first and then decreased. When the antibody concentration was 0.6 µg/mL, the RLUmax/IC_50_ reached the maximum, while the IC_50_ was small. Therefore, the optimal concentration of antibody was determined as 0.6 µg/mL.

### 3.3. Optimization of Pre-Incubation Time

**Figure 3:**
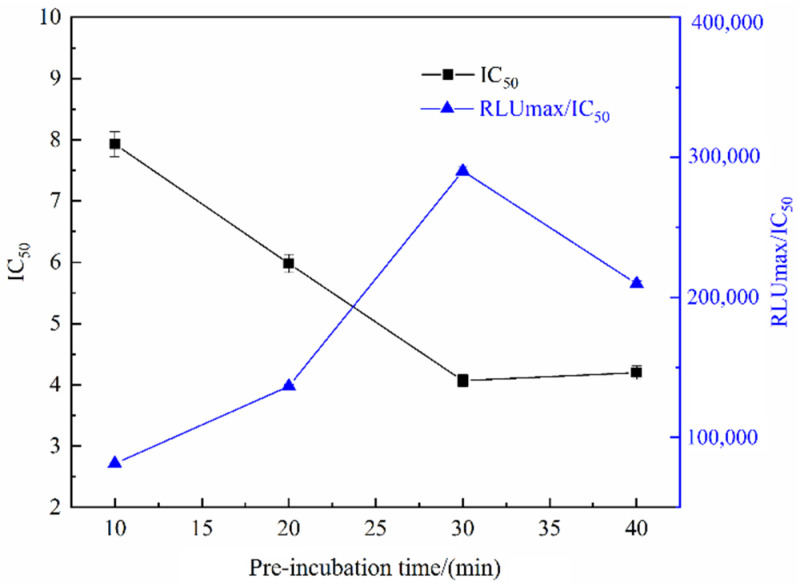
The IC_50_ and RLUmax/IC_50_ of ic-CLIEA with different pre-incubation time between ACE and its mAb for ACE detection.

In ic-CLIEA detection, the full binding between antigen and antibody is related to the pre-incubation time of them, which has great impact on the sensitivity of the detection. Therefore, the pre-incubation time of anti-ACE monoclonal antibody and ACE solution should be optimized. As shown in Figure 3, when the pre-incubation time was 30 min, RLUmax/IC_50_ was largest, while the IC_50_ was the smallest, so the optimal pre-incubation time between ACE and its mAb was determined to be 30 min.

### 3.4. Optimization of the Dilution Ratio of Goat Anti-Mouse-HRP Antibody

**Figure 4:**
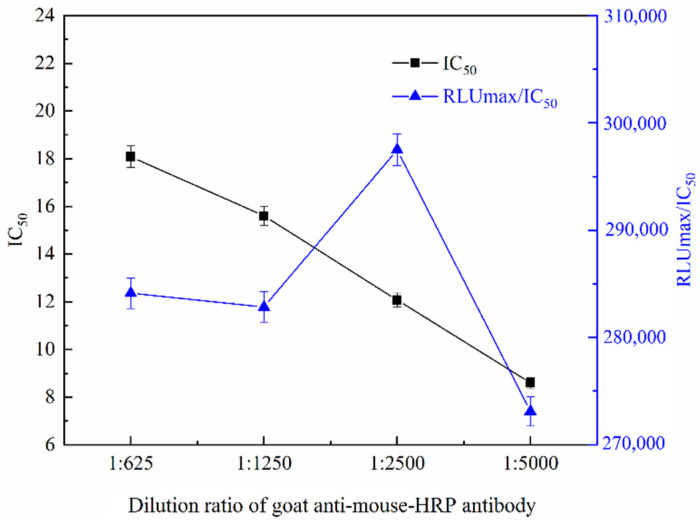
The IC_50_ and RLUmax/IC_50_ of ic-CLIEA with different goat anti-mouse-HRP antibody dilution ratios for ACE detection.

The effect of goat anti-mouse HRP secondary antibody concentration on the sensitivity of ic-CLEIA was further investigated. Goat anti-mouse HRP secondary antibody (1 mg/mL) was diluted by 1:625, 1:1250, 1:2500, and 1:5000, respectively, and then determined by ic-CLIEA. As shown in Figure 4, with the increase in the dilution ratio of goat anti-mouse HRP secondary antibody, the RLUmax/IC_50_ increased first and then decreased, while the IC_50_ gradually decreased. When the dilution ratio of the secondary antibody was 1:2500, the RLUmax/IC_50_ was the maximum, while the IC_50_ was small. Therefore, dilution of 1:2500 was selected as the optimal dilution ratio of goat anti-mouse HRP secondary antibody.

### 3.5. Optimization of Chemiluminescence Reaction Time

**Figure 5:**
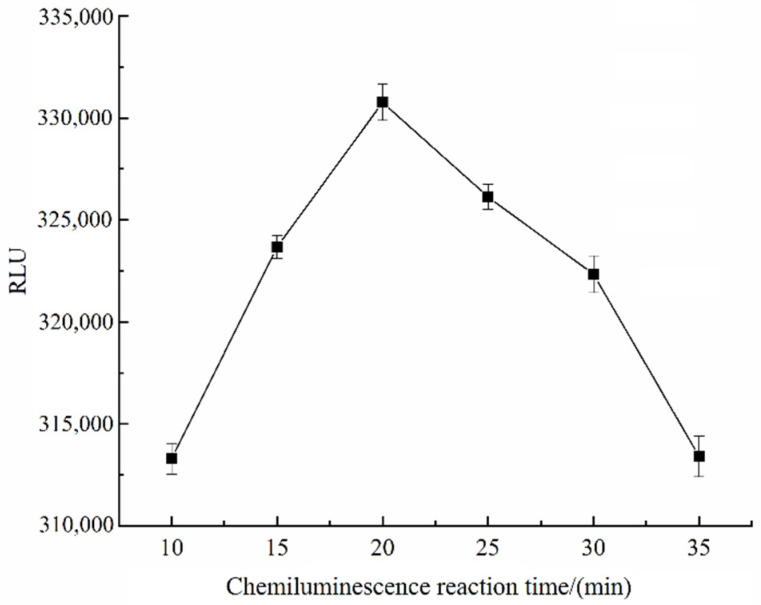
RLU values at different chemiluminescence reaction time.

In ic-CLIEA detection, the luminescence intensity first increased and then decreased with the extension of time after the addition of luminescent substrate, thus the optimal reaction time of chemiluminescence must be selected to ensure the sensitivity and accuracy of the experiment [18]. The reaction time of chemiluminescence substrate was set as 10, 15, 20, 25, 30, and 35 min, respectively, and the luminescence intensity of each group was measured. The results showed that the luminescence intensity reached the maximum value at 20 min, so the optimal reaction time of chemiluminescence was determined to be 20 min (Figure 5).

### 3.6. The Standard Curve of ic-CLIEA

**Figure 6:**
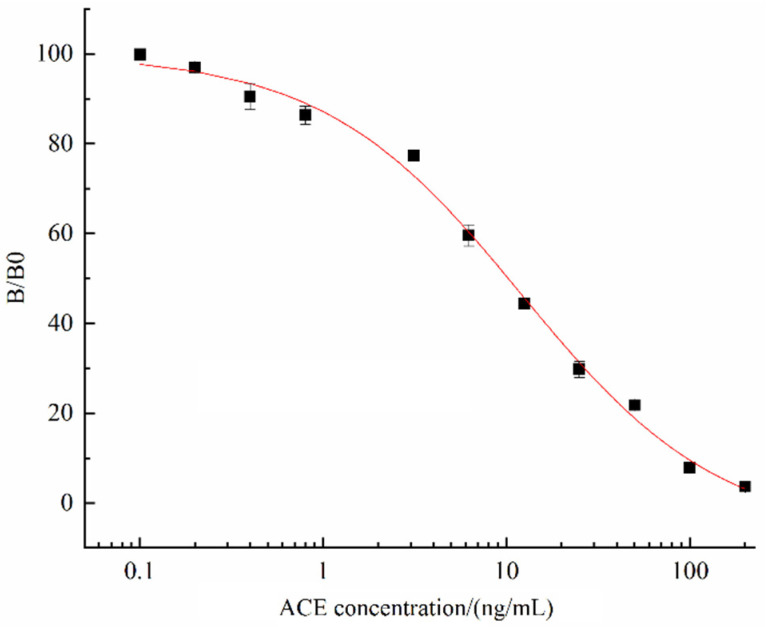
Standard curve of ic-CLIEA for ACE detection.

Based on the optimal ic-CLIEA detection conditions, the ACE concentration was taken as the abscissa and the B/B0 value as the ordinate, and the standard curve of ACE was drawn and fitted by Origin 2018. As shown in Figure 6, the standard curve equation was y = 7.11 + 107.09/[1 + (x/12.09)^0.80^], R^2^ = 0.995. According to the standard curve, the IC_50_ was 10.24 ng/mL, the detection range (determined as the IC_10_–IC_90_ according to the standard curve) was 0.70–96.31 ng/mL, and the LOD (IC_10_) was 0.70 ng/mL. According to the pretreatment and dilution method in this study, the LOD of ACE in actual sample was calculated to be 1.26 µg/kg.

### 3.7. Specificity of ic-CLIEA

Four structural analogues of neonicotinoids were selected for cross-reactivity determination, and the results were shown in Table 1. The cross-reactivity rates of ic-CLIEA for nitenpyram, thiacloprid, thiamethoxam, and clothianidin were all less than 10%, indicating that the ic-CLIEA had good specificity.

### 3.8. Elimination of Matrix Interference

**Figure 7:**
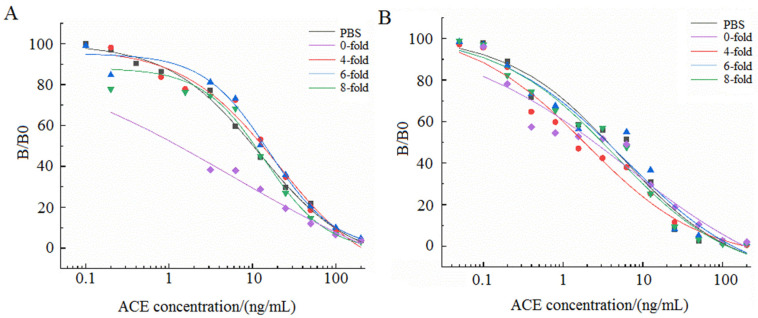
Matrix effect of Chinese cabbage (**A**) and cucumber (**B**) after dilution 0, 4, 6, and 8 fold with PBS solution.

In the process of immunoassay, it is crucial to eliminate matrix interference, because the pH, ionic strength, and organic matter content of real samples will interfere with the specific reaction between antigen and antibody, thus affecting the sensitivity of detection [21]. In this experiment, the extract juice of Chinese cabbage and cucumber were diluted 0, 4, 6, and 8 fold with PBS solution, respectively. Subsequently, standard curves drawn with the diluted solution of the matrix juice were compared with those drawn with PBS, and the appropriate dilution ratio was chosen to eliminate matrix interference. Figure 7 shows that the extract juice of Chinese cabbage and cucumber has little influence on the standard curve after 6-fold dilution, so the 6-fold dilution of the sample extract juice was chosen for the determination of ACE in the subsequent test.

### 3.9. Recovery Experiments

According to the LOD (the LOD in real samples was 1.26 µg/kg) and the detection range (the detection range in real samples was 1.26–173.36 µg/kg) of the ic-CLIEA, the added concentration of ACE in real samples was set as 1.5, 6, and 30 μg/kg. As shown in Table 2, the average recovery rate of ACE in Chinese cabbage and cucumber determined by ic-CLIEA was 82.7–112.2% with CV less than 9.19%, and the average recovery rate of ACE determined by HPLC was 80.7–118.00% with CV less than 9.08%. The results showed that the established ic-CLIEA method was accurate and reliable, and had a good correlation with HPLC, which could be used for the detection of ACE residue in vegetables.

### 3.10. Comparison of Some Published Results for ACE Rapid Detection

In recent years, many rapid methods for ACE residue detection have been reported. Comparing the results of this research with the published results, it was shown that the ic-CLIEA method established in this study does not require the synthesis of any materials, the detection materials and reagents are easily available, the pretreatment and operation procedures are simple, as well as has a wide linear range and a low detection limit, which is suitable for ACE detection (Table 3).

## 4. Discussion

In the immunoassay, the sensitivity and stability of the method mainly depend on the balance of the specific reaction and reversibility reaction between antigen and antibody [29]. Therefore, a series of conditions affecting the sensitivity of ic-CLIEA were optimized in this study, including the concentration of coating antigen, antibody concentration, the pre-incubation time of anti-ACE monoclonal antibody and ACE (sample) solution, the dilution ratio of goat anti-mouse HRP antibody, and chemiluminescence reaction time. The results showed that the concentrations of antigen and antibody were the key factors affecting the reaction balance. If the concentrations of antigen and antibody are too low, the reaction is not complete, and if the concentrations of antigen and antibody are too high, it is easy to cause multilayer adsorption, which leads to the mutual cover of the antigenic determinants, thus affecting the stability and sensitivity of the analytical method. In addition, the reaction time of antigen and antibody is also an important factor affecting the sensitivity and accuracy of the method. With the extension of the reaction time, the IC_50_ of the method shows a trend of decreasing first and then increasing, which may be because too short a reaction time will lead to incomplete binding between antigen and antibody, and too long a reaction time will easily cause non-specific adsorption. Therefore, only appropriate reaction time can make the sensitivity of the method reach the best. Matrix effect refers to the non-specific reaction to the substance in the extract during immunoassay, which may lead to inaccurate results in actual sample analysis. Usually, matrix effects can be eliminated by simple dilution prior to analysis. The dilution ratio at which there is no significant difference between the absorbance of the extract solution with or without sample matrix should be confirmed to manage the matrix effect [30].

Finally, a highly sensitive ic-CLIEA for ACE detection was successfully established, with an IC_50_ of 10.24 ng/mL, a detection range (IC_10_–IC_90_) of 0.70–96.31 ng/mL, and a LOD (IC_10_) of 0.70 ng/mL (according to the pretreatment and dilution method in this study, the LOD in real samples was 1.26 µg/kg). Although the LOD of the established ic-CLIEA in this study is not the lowest among all reported methods, the materials and reagents used in this method are easily available, the accuracy and selectivity are high, the detection sensitivity can meet the MRL requirements of ACE in vegetables, and the quantitative detection can be achieved. Most importantly, the pretreatment method is very simple and suitable for rapid high-throughput screening of ACE residues in vegetables, which has a good application prospect. In addition, the detection method established in this study is also suitable for the rapid detection of other compounds and pesticides, but the experimental parameters need to be re-optimized to improve the detection sensitivity.

## Figures and Tables

**Table 1:** Cross-reactivity of ACE and its analogues with anti-ACE mAb determined by ic-CLIEA (*n* = 3).

Pesticide	Structural Formula	IC_50_ (ng/mL)	Cross-Reactivity
acetamiprid	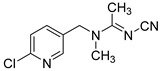	10.24	100
nitenpyram	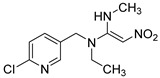	522.45	1.96
thiacloprid	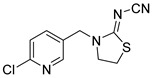	213.78	4.79
thiamethoxam	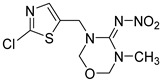	>10,000	<0.1
clothianidin	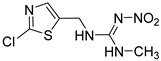	118.66	8.63

**Table 2:** Recoveries of acetamiprid in Chinese cabbage and cucumber (*n* = 3).

Sample	Spiked Level/(μg/kg)	ic-CLIEA			HPLC		
		Found/ (μg/kg)	Average Recovery/%	CV/%	Found/ (μg/kg)	Average Recovery/%	CV%
Chinese cabbage	1.5	1.24	82.7	2.71	1.77	118.0	4.97
	6	5.54	92.3	5.68	6.70	111.7	9.08
	30	33.67	112.2	8.48	31.71	105.7	6.10
Cucumber	1.5	1.29	86.0	9.19	1.21	80.7	3.21
	6	5.14	85.7	4.01	4.86	81.0	2.01
	30	29.78	99.3	4.20	28.89	96.3	0.80

**Table 3:** Comparison of some published results for ACE detection with this research.

Methods	Synthesis of Materials	Linear Range (ng/mL)	LOD (ng/mL)
lateral flow assay [22]	no need	no	1
lateral flow assay [23]	AuNPs@polyA-cDNA	no	0.33
colorimetric [24]	gold nanoparticles (AuNPs)	5567–66803	848
surface-enhanced Raman [25]	Ag-coated cellophane	no	1000
colorimetric and fluorescence [26]	AuNPs	5.56–222	0.08
fluorescence [27]	Cationic carbon dots (cCDs)	0.357–26.8	0.067
chemiluminescence sensor [28]	graphene oxide (GO) and AuNPs	0.0047–2	0.002
this work	no need	0.70–96.31	0.70

## Data Availability

The data presented in this study are available on request from the corresponding author.
